# MicroRNA Profiling Identifies Diagnostic and Prognostic Markers in Pediatric Sarcoma

**DOI:** 10.3390/cancers17233791

**Published:** 2025-11-27

**Authors:** Terrie G. Flatt, Leonid M. Yermakov, Shreeram Akilesh, Eleanor Y. Chen, Elizabeth Gonzalez, Alejandro Parrales, Marta Zapata-Tarres, Rocio Cardenas-Cardos, Liliana Velasco-Hidalgo, Celso Corcuera-Delgado, Rodolfo Rodriguez-Jurado, Lillia García-Rodríguez, Midhat S. Farooqi, Atif Ali Ahmed

**Affiliations:** 1 Department of Pediatrics, Division of Hematology & Oncology, Children’s Mercy Hospital, University of Missouri, Kansas City, MO 64110, USA; tgflatt@cmh.edu (T.G.F.); eliglezdgz@gmail.com (E.G.); aparralesbriones@cmh.edu (A.P.); 2 Department of Laboratory Medicine and Pathology, University of Washington, Seattle, WA 98195, USA; yermakov@uw.edu (L.M.Y.); shreeram@uw.edu (S.A.); eleanor2@uw.edu (E.Y.C.); 3 MD/PhD (PECEM) Program, Facultad de Medicina, Universidad Nacional Autónoma de México, Mexico City 04360, Mexico; 4 Research Coordination Mexican Institute of Social Security Foundation, Mexico City 06600, Mexico; mzapatatarres@gmail.com; 5 Departamento de Oncología Pediátrica, Instituto Nacional de Pediatría, Mexico City 04530, Mexico; oncoped_inp@hotmail.com (R.C.-C.); lilianavh@hotmail.com (L.V.-H.); 6 Departamento de Patología Pediátrica, Instituto Nacional de Pediatría, Mexico City 04530, Mexico; ctcorcuera@hotmail.com (C.C.-D.); rrrj60@hotmail.com (R.R.-J.); 7 Facultad de Medicina, Universidad de Monterrey, San Pedro Garza García, Monterrey 66238, Mexico; legr.29@gmail.com; 8 Department of Pathology & Laboratory Medicine, Children’s Mercy Hospital, University of Missouri, Kansas City, MO 64110, USA; msfarooqi@cmh.edu; 9 Department of Pathology and Laboratory Medicine, Seattle Children’s Hospital, Seattle, WA 98105, USA

**Keywords:** microRNA, rhabdomyosarcoma, Ewing’s sarcoma, osteosarcoma, NanoString nCounter, microRNA in situ hybridization

## Abstract

Background: MicroRNAs (miRNAs) function as post-transcriptional gene expression regulators and influence the development and progression of several cancers, yet their roles in pediatric sarcomas remain poorly defined. Methods: RNA extracted from formalin-fixed paraffin-embedded tumor tissue scrolls of 108 pediatric tumors, including 32 osteosarcoma (OS), 26 Ewing’s sarcoma (EWS), and 50 rhabdomyosarcoma (RMS) cases, were analyzed for microRNA expression using the NanoString multiplex nCounter platform that yielded information on 827 human miRNAs. The expression of candidate miRNAs was validated with in situ hybridization (miRNA-ISH) and QuPath quantification on tissue microarray slides comprising an independent set of 48 OS, 17 EWS, and 104 RMS adult and pediatric cases collectively. Results: The differential expression analysis of nCounter data identified 23 miRNAs enriched in RMS, 33 in EWS, and 45 in OS (>3 fold change and *p* < 0.01). miR-206 was most strongly associated (>55 fold change, *p* < 1 × 10^−9^) with RMS and demonstrated the highest sensitivity and specificity in distinguishing RMS from EWS and OS; this finding was also confirmed by miRNA-ISH. A combined signature of differentially expressed miRNAs reliably separated alveolar from embryonal RMS. The expression of miR-9-5p in EWS and miR-140-5p in OS discriminated among the different tumors and correlated with adverse patient outcome. The nCounter assay exhibited greater sensitivity than miRNA-ISH in detecting miR-206 and miR-140-5p expression. Conclusions: Collectively, these findings demonstrate that distinct miRNA profiles can differentiate pediatric sarcoma types and subtypes and offer clinically relevant insights into tumor biology, prognosis, and potential diagnostic application.

## Simple Summary

MicroRNAs are small non-coding RNA molecules that play a significant role in many physiological processes in the body, including the regulation of gene expression. Their expression in cancer cells can become dysregulated to the point where they promote tumorigenesis or function as tumor suppressors. In this article, we define the microRNA profile associated with three common pediatric sarcomas. Using multiple tissue samples from different sources including tissue microarray slides and two methods, we have detected the differential expression of miR-9-5p, miR-206, and miR-140 in Ewing’s sarcoma, rhabdomyosarcoma, and osteosarcoma, respectively. The NanoString nCounter profiling method exhibited higher sensitivity in detecting microRNA profiles and differentially expressed microRNAs compared with microRNAscope which identified the in situ hybridization of specific microRNA molecules. MicroRNA expression correlated with adverse patient outcome. Our findings demonstrate that distinct miRNA profiles can differentiate pediatric sarcoma types and provide clinically relevant insights into potential diagnostic and prognostic applications.

## 1. Introduction

MicroRNAs (miRNAs) are small non-coding RNAs that control key physiologic processes including embryonic development and cellular proliferation, differentiation, metabolism, and apoptosis. They are known to regulate post-transcriptional expressions and functions of target genes by degrading mRNA, altering gene expression, and/or modifying protein translation [1,2]. The activation and function of miRNAs are spatially and temporally regulated in response to various physiologic and pathologic stimuli and are frequently dysregulated in different disease states including cancer [2,3]. Dysregulated miRNAs contribute to cancer pathogenesis and tumor growth through their interaction with signaling and apoptosis pathways. They exhibit unique tissue-specific and tumor-specific expression profiles that can accurately determine tumor origin and distinguish malignant from normal tissue as well as among different cancer types [4,5,6]. The profiling of dysregulated miRNA expression has proven valuable for tumor classification, diagnosis, and prognosis [4,5]. miRNA-based therapeutic approaches are currently in preclinical development as a cancer treatment strategy and include mimics that simulate miRNA tumor suppressive properties or anti-miRs that inhibit oncogenic miRNAs [6].

Only a few publications have highlighted the role of miRNAs in pediatric tumors, where they contribute to carcinogenesis and affect clinical outcomes. A preliminary study has identified specific miRNA profiles in association with several pediatric malignancies [7]. Pediatric cancers sometimes share overlapping morphologic features and such profiles may help in their accurate diagnosis and management. Differential miRNA expression may also help identify signatures associated with aggressive behavior or adverse prognosis. The study of miRNAs has recently been facilitated by Nano String digital barcoding technology which allows for miRNA quantification from formalin-fixed paraffin-embedded (FFPE) tissue. The NanoString nCounter profiling platform counts the actual numbers of mature miRNA transcripts that are captured with fluorescently barcoded probes and has been used to retrieve miRNA in low concentrations [8]. A novel RNA in situ hybridization (ISH) technology with unique probes designed to detect specific miRNAs allows for chromogenic readout, bright-field detection, and the semi-quantification of miRNAs on tissue sections, enabling correlative spatial analysis [9]. Unlike RT-PCR, both methods obviate the need for nucleic acid amplification and are optimized on FFPE tissue. In this study, we evaluate miRNA expression in a large cohort of Ewing’s sarcoma (EWS), osteosarcoma (OS), and rhabdomyosarcoma (RMS) tumor cases. Our results have uncovered distinct tumor specific profiles and differential expression patterns that may offer tremendous value in tumor classification and subtyping. Moreover, distinct miRNA signatures may be associated with metastasis and adverse clinical outcomes, highlighting their potential utility in prognostic classification and personalized treatment strategies.

## 2. Materials and Methods

### 2.1. Patients and Materials

NanoString nCounter profiling was performed on pediatric (ages 0–18 years) patients who were diagnosed and treated for EWS, OS, and RMS at Children’s Mercy Hospital (CMH) in Kansas City, MO, USA, and the Instituto Nacional de Pediatría (INP) in Mexico City, Mexico, between May 2012 and May 2019. RNA was extracted from archived formalin-fixed paraffin-embedded (FFPE) tumor blocks. Overall, 108 tumor cases were selected including 32 OS, 26 EWS, and 50 RMS (22 alveolar and 28 embryonal subtypes). The pathologic diagnosis was confirmed after reviewing the histology, immunophenotype, and pertinent molecular features. Cases from diagnostic or initial biopsies were included, some of which underwent histological decalcification prior to processing. Histology blocks with the highest viable tumor cell density (more than 70% tumor nuclei) and lowest proportion of stromal and inflammatory cells were selected for scroll sectioning. Tissue blocks with tumor necrosis or insufficient viable tumor proportion were excluded. Patients received standard chemotherapy protocols and had variable follow-up periods, where outcomes were categorized as alive or deceased from progressive disease. Additional clinical information including the presence or absence of metastasis was recorded (Table 1).

MicroRNA ISH validation studies were performed on tissue microarray (TMA) slides obtained from separate sources. Commercial Osteosarcoma (OS 804d) and Rhabdomyosarcoma (SO2082b) TMA slides were purchased from Tissue Microarray Inc. (Derwood, MD, USA) containing 40 OS and 96 RMS adult and pediatric cases. Additional TMA slides obtained from the department of Laboratory Medicine and Pathology, University of Washington, Seattle, WA, USA, contained 9 duplicate archived EWS cases from patients older than 18 years. Multi-tumor pediatric TMA slides containing 8 EWS, 8 OS, 5 fusion-positive alveolar RMS (ARMS), and 3 embryonal RMS (ERMS) archived cases were obtained from Seattle Children’s Hospital, Seattle. Local TMA blocks were previously constructed from three-millimeter tissue cores of viable tumor collected between 2019 and 2023.

### 2.2. NanoString nCounter miRNA Profiling

RNA was retrieved from de-identified FFPE tumor tissue scrolls from pediatric hospital patients (n = 108 including RMS 50; EWS 26; OS 32) using the Qiagen miRNeasy FFPE kit. Extracted RNA had RIN values of 1.1–2.7 and was deemed of sufficient quantity and quality to be processed for the NanoString multiplex platform (nCounter Human v3 miRNA Expression Assay kit), according to previously described methods [9]. An input of 100 ng of RNA was loaded into the assay. The expression of 827 human miRNAs has been sequenced with high confidence and analyzed for clinically relevant miRNAs. NanoString nCounter processes discrete counts of measurements similar to RNA-seq and uses similar normalization methods to filter out low-expression results [10]. Stringent normalization of miRNA data was performed by eliminating low digital counts and counts with high variability. Positive control and codeset normalization were then performed using the geometric mean algorithm to create a normalization factor. Normalized data of the top 100 miRNA transcripts was analyzed using ROSALIND^®^ software, with a HyperScale architecture developed by ROSALIND, Inc. (San Diego, CA, USA). Read distribution violin plots, identity heatmaps, and sample MDS plots were generated as part of the QC step. The limma R library was used to calculate fold changes and *p*-values and perform optional covariate correction [11]. Clustering of differentially expressed miRNA was performed using the PAM (Partitioning Around Medoids) method using the fpc R library [12]. The top targeted gene predictions, validated genes, and related drugs and diseases were analyzed using the multiMiR R library (V2.4) [13]. miRNA secondary structures were calculated and visualized using the ViennaRNA software (V 2.7.0) [14]. All tumor samples have passed QC thresholds except for 3 EWS, 2 RMS, and 2 OS cases that did not meet stringent normalization. The remaining samples (23 EWS, 48 RMS, and 30 OS) were processed for analysis. Tumors were compared for their miRNA expression profiles using fold change and *p*-values.

### 2.3. MicroRNA-Scope

In situ hybridization for miRNA (miRNA-ISH) was completed on the FFPE TMA slides using the miRNAscope^TM^ HD assay kit (Advanced Cell Diagnostics, Newark, CA, USA). The slides were baked for 1 h at 60 °C, deparaffinized in xylene, dipped in 100% ethanol, and dried at 60 °C. Slides were post-fixed overnight in 10% neutral-buffered formalin, rinsed in water, and dried. Hydrogen peroxide was added for 10 min to quench endogenous peroxidase activity. Slides underwent a 15 min target retrieval solution incubation at 100 °C and Protease 3 incubation for 30 min at 40 °C. Following pretreatment, the miRNAscope^TM^ detection assay was completed according to manufacturer directions using probes from the same manufacturer. The slides were counterstained with 50% Gill 1 hematoxylin for 2 min and cover-slipped with EcoMount (Biocare, Pacheco, CA, USA). Experiments included negative and positive hybridization controls and three target probes for miR-9-5p, miR-206, and miR-140 (Supplementary Table S1). Hybridization signals were recorded as red spots and quantified using the QuPath software (V 0.5.1).

### 2.4. QuPath Quantification

Probed TMA slides were scanned with Aperio CS2 digital scanner and the virtual slides (.svs) were analyzed for positive ISH signals using QuPath v0.5.1 [15]. Stain color deconvolution was performed using a representative region of interest (ROI) for hematoxylin and an miRNA probe. Cell detection was achieved using the default hematoxylin OD method with minor adjustments of nucleus and intensity parameters. Subcellular miRNA probe detection was performed with an expected probe spot size of 0.5 μm^2^ (0.2–0.8 μm^2^ range). Cells were classified as positive with 1–10 estimated number of hybridization spots; cases with greater than 10 spots were labeled as high expression. Negligible differences in probe intensity were noted between batches (positive RNU6 probe control was specifically repeated on each slide for inter-batch comparison purposes) with only minor color vector adjustments performed to minimize inter-experiment variability. Tissue cores with a complete absence of positive control probe staining and a high degree of tissue artifacts, such as tissue folding, disruption, or pigmentation, were excluded, rendering the total number of interpretable cases as 90 RMS, 14 EWS, and 42 OS. Scramble probe-stained tissues were reviewed and verified to have no positive background signal.

### 2.5. Statistical Analysis

Fold changes and adjusted *p*-values for nCounter differential expression comparisons were generated with an integrated statistical software package. *p*-value adjustment was performed using the Benjamini–Hochberg (BH) method for estimating false discovery rates. All nCounter cases were included in the measurements of sensitivity and specificity, which were calculated with MedCalc^®^ Statistical Software version 20.214 (MedCalc Software Ltd., Ostend, Belgium). QuPath statistical analysis for miRNA-ISH was performed in RStudio (Posit) using R, version 4.4.1 (R Foundation for Statistical Computing, Vienna, Austria) [16,17]. The tidyverse package was used for data tidying, manipulation, and visualization [18]. ROC curves and normalized H-scores were calculated for each probe.

## 3. Results

**Figure 1:**
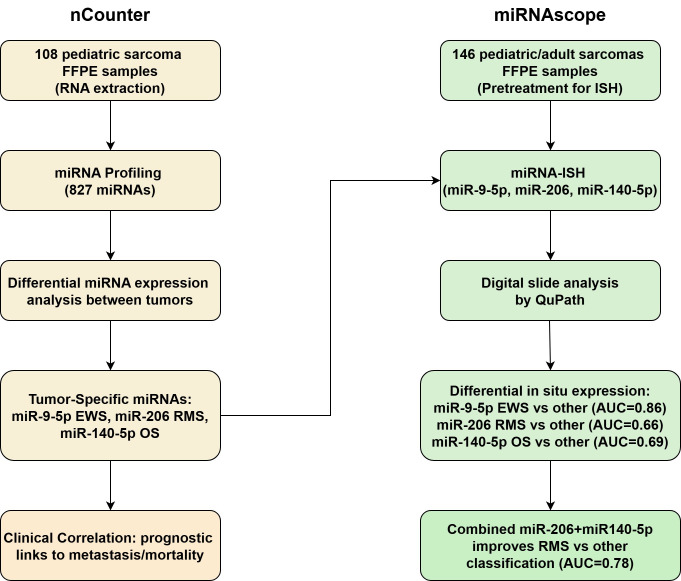
Profiling workflow. NanoString nCounter experiments highlighted differential expression of miR206, miR-9-5p, and miR-140-5p in an initial sample of 108 pediatric sarcomas. miRNAscope experiments revealed in situ hybridization of the three selected miRNAs in multi-tumor microarray slides.

**Figure 2:**
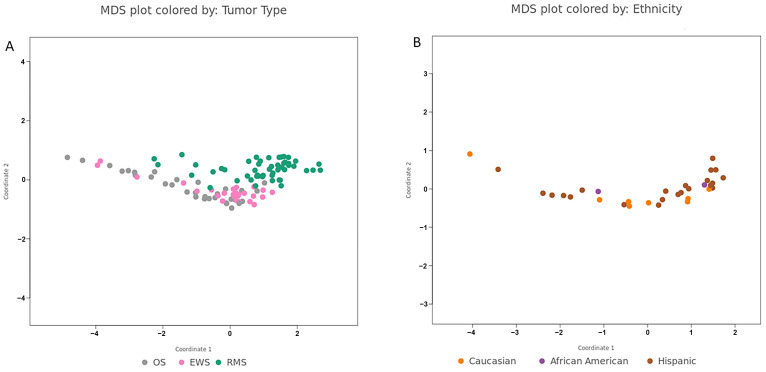
miRNA MDS dot cluster analysis. miRNA expression revealed segregated clusters based on the tumor type (**A**) and exhibited overlap when analyzed according to patients reported ethnicity (**B**).

**Figure 3:**
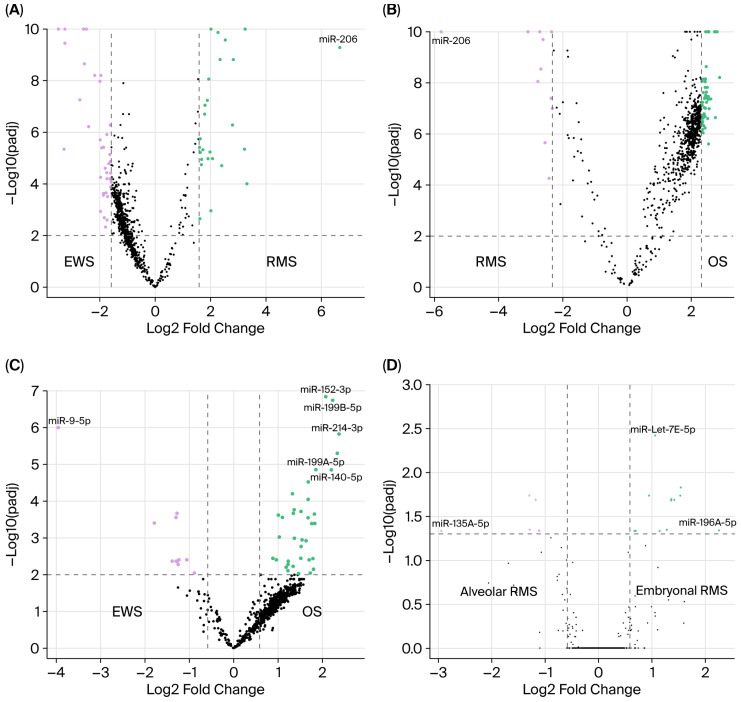
NanoString nCounter profiling revealing miRNA differential expression between RMS and EWS (**A**), between OS and RMS (**B**), between EWS and OS (**C**), and between ARMS and ERMS (**D**). Green and purple dots represent significantly expressed miRNA in each respective tumor. Horizontal and vertical dashed lines represent statistical significance threshold values for *p*-value and fold-change, respectively. Black dots represent miRNA expression that did not reach statistical significance. miR-206 had the most significant expression in RMS compared with EWS [log2 fold change 6.66197, −log10 *p* = 9.28567] and OS [log2 fold change 5.79177, −log10 *p* = 10.54212]. miR-9-5p expression was identified in EWS [log2 fold change 3.95510, −log10 *p* = 6.00218]. Although miR-140-5p expression in OS was not the most significant, it provided more consistent differential results than other miRNAs. Differential expression of miRNAs in alveolar versus embryonal RMS included Let-7e and miR-196A in embryonal RMS and miR-135A-5p in alveolar RMS.

**Figure 4:**
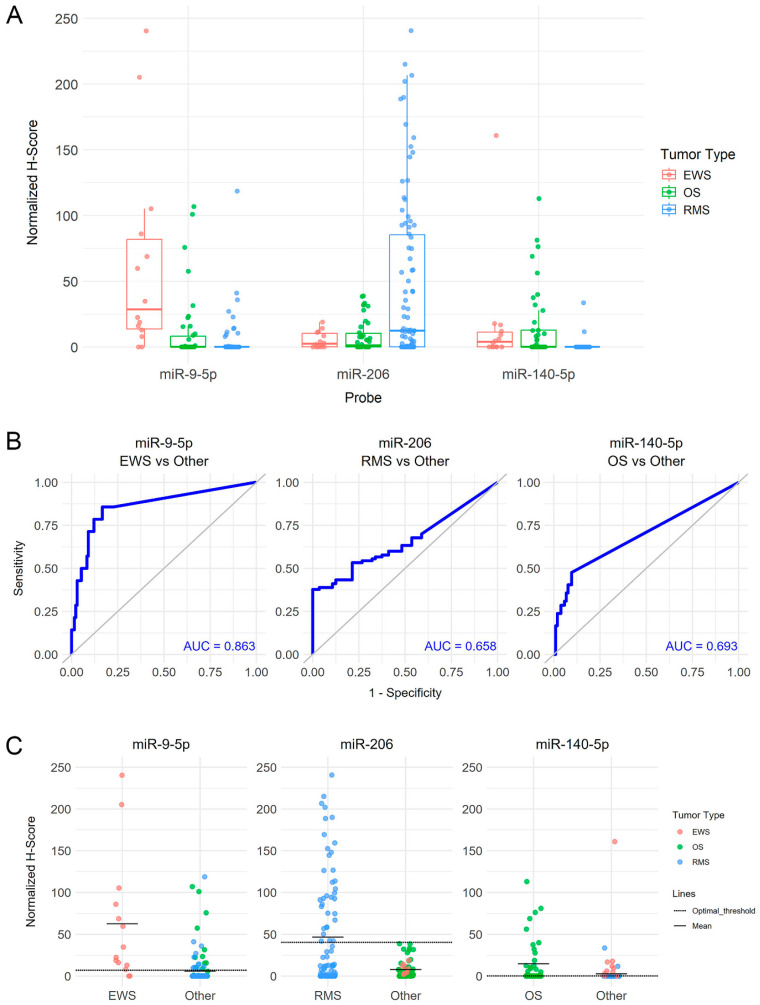
miRNA-ISH experiments revealing the staining scores of miR-9-5p, miR-206, and miR-140-5p in tissue microarray slides of EWS, OS, and RMS represented as box plots (**A**). Performance of each probe staining in detecting specific tumors from the others is represented in the AUC sensitivity–specificity graphs (**B**) and normalized H-staining scores (**C**).

The microRNA profiling experiment workflow is summarized in Figure 1. Tumors segregated separately in nCounter multidimensional scaling, indicating distinct miRNA profiles (Figure 2). This separation was particularly pronounced for RMS. In contrast, miRNA clusters corresponding to the patients’ age groups, sex, and reported racial backgrounds (Table 1) demonstrated an overlap suggesting a lack of correlation between tumor miRNA expression and these parameters. The differential expression of miRNA, including 23 in RMS, 33 in EWS, and 45 in OS, was identified in the nCounter platform, corresponding to genes that are mostly involved in tumor differentiation and carcinogenesis (Table 2). miR-9-5p, miR-206, and miR-140 exhibited the strongest association with EWS, RMS, and OS, respectively (Figure 3A–C). These miRNAs were further selected for miRNA-ISH experiments which validated their differential expression (Supplementary Table S1). Receiver operating characteristic (ROC) analysis and Youden’s J statistic were performed to assess the diagnostic utility of miRNA-ISH in tumor classification (Figure 4).

### 3.1. miRNA Expression in Rhabdomyosarcoma

**Figure 5:**
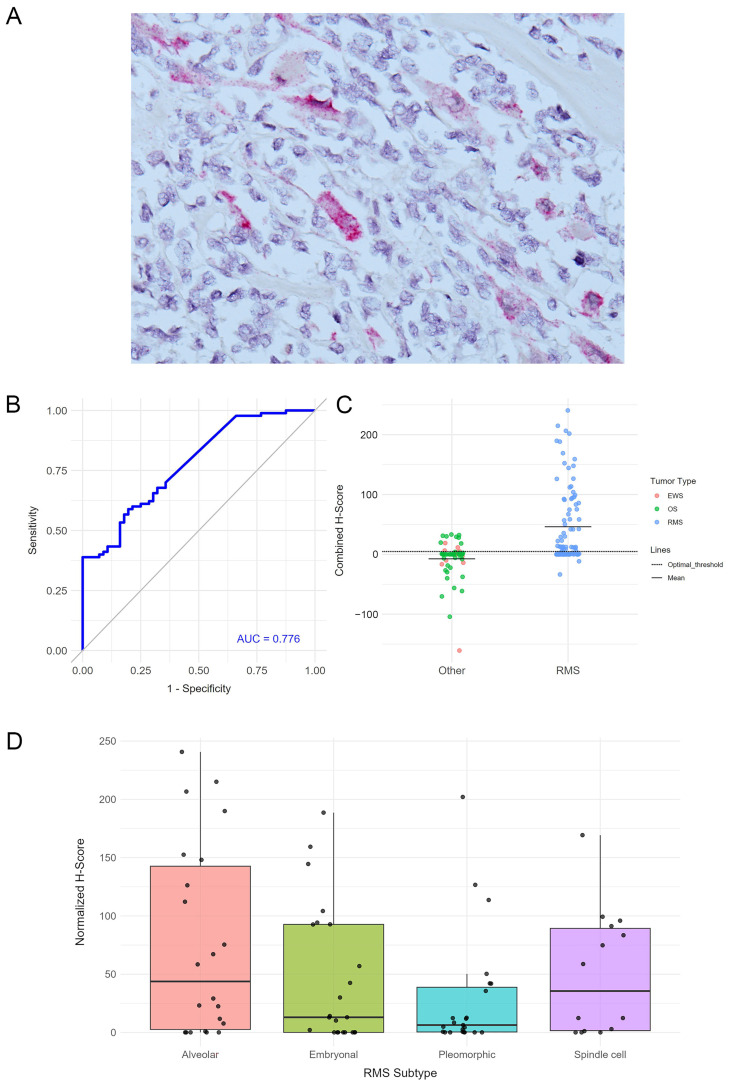
Performance of miR-206 in situ hybridization in RMS and its types. In situ hybridization is visible as eosinophilic cytoplasm dots in tumor cells (**A**). Combined expression values of miR-206 and miR-140-5p exhibited a better diagnostic performance with higher AUC curve values (**B**) and H-score dot plots (**C**) than miR-206 alone. MiR-206 hybridization is higher in ARMS but with no significant differences from the other RMS types (**D**). Magnification ×400 (**A**).

RMS exhibited a unique miRNA profile that was distinct from OS and EWS, with the significant expression of miR-206, miR-450A-5p, and miR-483-3p, in contrast to the expression of miR-29a-3p, miR-221-3p, miR-497-5p, and miR-195-5p in EWS and miR-140-3p, miR-181A-3p, and miR-218-5p in OS (Figure 3). miR-206 had the largest differential expression in RMS compared with EWS (101.26-fold change, *p* = 5.18 × 10^−10^) and OS (55.4-fold change, *p* = 2.87 × 10^−11^) and exhibited the highest sensitivity and specificity in distinguishing RMS from the other tumors (AUC 0.95). nCounter reading counts ranged from 9.87040 to 17.46250, with counts >9.7 yielding a sensitivity of 98% and specificity of 83% for detecting RMS against EWS and OS (confidence interval: 70.6–91.4). Raising the expression count threshold to >14.0 increased the specificity to 100% for classifying a tumor as RMS (Supplementary Table S2). According to miRNA-ISH, the miR-206 signal was detected in 34/90 (38%) of RMS and was absent in EWS and OS cases, corresponding to 100% specificity. However, because of the lower sensitivity, miR-206 had a predictive AUC value of 0.66 (Figure 3). On the other hand, the miR-140-5p ISH signal was detectable but significantly reduced in RMS compared with both OS (*p* = 0.0004) and EWS (*p* = 0.01). Combining miR-206 and miR-140-5p ISH expression values, achieved by subtracting the normalized H-score of miR-140-5p from that of miR-206, yielded an improved sensitivity and specificity for differentiating RMS with an AUC of 0.776 (Figure 5).

Within RMS subtypes, the nCounter data has identified 18 miRNAs differentially expressed between ARMS and ERMS (≥1.5-fold change and *p* < 0.05). let-7e-5p (2.08-fold change, *p* = 0.00379) and miR-196A-5p (4.8-fold change, *p* = 0.05) were the most significantly expressed in ERMS. Out of six miRNAs, miRNA 135A-5p had the largest fold change in alveolar RMS (7.7-fold change, *p* = 0.05) (Figure 3D). Although any of these miRNAs can differentiate between ARMS and ERMS (Table 3), combination signatures of several miRNA provided superior discriminatory power. For example, combining high miRNA 135-5p expression count >8.23084 with low let-7r-5p expression counts ≤8.34993 yielded a sensitivity of 75% and specificity of 80% for alveolar RMS detection. miR-206 ISH demonstrated higher expression in alveolar RMS but without significant differences from other RMS types (Figure 5D).

### 3.2. miRNA Expression in Ewing’s Sarcoma and Osteosarcoma

A total of 47 miRNAs were differentially expressed between EWS and OS (>1.5-fold change and *p* < 0.01) including 11 in EWS and 36 in OS (Table 2). Among these, miR-214-3p, miR-193, miR-199B-5p, miR-140-5p, and miR-152-3p had the most significant expression in OS while miR-9-5p and miR-29 were prominent in EWS (Figure 3). nCounter reading counts of miR-9-5p in EWS ranged from 5.79227 to 14.09050 (Supplementary Table S2). A reading count threshold >9.0 yielded a sensitivity of 73% (CI: 52–88%) and specificity of 74% (CI: 63–83%) for detecting EWS. miRNA-ISH confirmed miR-9-5p ISH expression in 12 of 14 (86%) EWS, 11 of 90 (12%) RMS, and 11 of 42 (26%) OS cases and demonstrated a stronger predictive accuracy for EWS with an AUC value of 0.86 (Figure 4). The normalized H-score for miR-9-5p was significantly lower in OS (*p* = 1.59 × 10^−7^) and RMS (*p* = 1.52 × 10^−10^) compared with EWS with no significant difference between OS and RMS (Figure 4).

miR-140-5p expression in OS nCounter cases differentiated OS from EWS and RMS (Figure 2) with nCounter reading counts ranging from 5.92265 to 13.3009 (Supplementary Table S2). A reading count threshold >7.0 yielded a sensitivity of 94% (CI 79–99) and specificity of 80% (CI: 69–88) for detecting OS against the other tumors. Interestingly, 10/26 EWS cases had reading counts that exceeded this threshold, thus explaining the lower specificity. miRNA-ISH validation confirmed higher expression in OS compared with RMS (*p* < 0.00001). Similarly, a substantial number of EWS cases had positive miR-140-5p expression rendering the difference between OS and EWS as not significant and explaining the relatively lower AUC predictive value of 0.69 (Figure 3 and Figure 4).

### 3.3. miRNA Expression and Patients’ Outcomes

**Figure 6:**
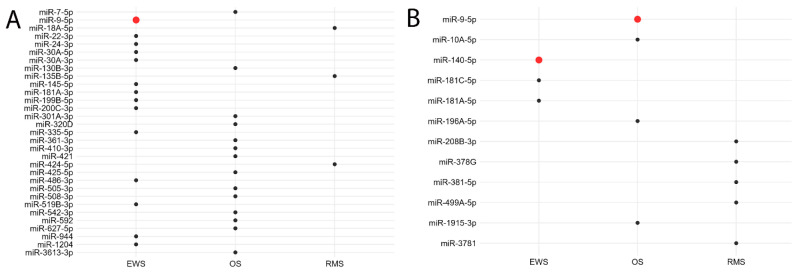
miRNA expression in relation to adverse outcome where each miRNA is represented as a circular dot. (**A**) Differentially expressed miRNAs in metastatic tumors. miR-9-5p is highlighted as a red dot in EWS. (**B**) Differentially expressed miRNAs in tumors from deceased patients. miR-140-5p and miR-9-5p are highlighted as red dots in EWS and OS, respectively. Although the differences were significant on unadjusted *p*-value calculations, they did not achieve the level of significance after stringent BH correction (adjusted *p* = 0.05–0.08).

Using the nCounter platform, several miRNAs were differentially expressed in association with distant metastasis and patient mortality. Thirteen miRNAs, including miR-9-5p, were enriched in metastatic EWS tumors, while fifteen miRNAs were associated with metastasis in OS and three in RMS (Figure 6A). A distinct set of miRNAs were differentially expressed in tumors from patients who died during follow-up. miR-9-5p and miR-140-5p were among those associated with mortality in OS and EWS patients, respectively (Figure 6B). Although the differences were significant on unadjusted *p*-value calculations, they did not remain significant after BH correction.

## 4. Discussion

There is a paucity of studies regarding microRNA differential expression in pediatric tumors. In one study, several dysregulated microRNAs could differentiate between neuroblastoma and RMS in xenograft animal models, with the overexpression of miR-7, miR-218, miR-137, miR124a, and miR-16 in neuroblastoma and miR-182, miR-133b, and miR-133a in RMS [7]. miR-1-1/miR-133a-2, miR-1-2/miR133a-1, and miR-206/miR-133b have been found to control the differentiation of myogenic precursor cells and may thus contribute to RMS development [19]. Specific miRNA profiles were found to be expressed in pediatric adrenocortical tumors, where they affected the prognosis and the 5-year survival rate [20]. Thus, miRNA profiles may be tumor-specific and reflect cancer-related function, whether it is tumor suppression or oncogenic stimulation. Along that line, our study has uncovered unique and distinct miRNA profiles in EWS, OS, and RMS. The pathologic distinction of these tumors may occasionally be challenging due to overlapping morphologies, uncommon anatomic locations, small tissue samples, and inconclusive molecular tests. Differential miRNA expression may aid in the diagnosis of pediatric sarcoma and provide prognostic insights for patients’ management, similar to findings in other cancers [7]. In this study we have profiled miRNA expression across pediatric rhabdomyosarcoma, Ewing’s sarcoma, and osteosarcoma. The NanoString nCounter results supported by miRNA-ISH have identified miR-206, miR-9-5p, and miR-140-5p as potential discriminatory biomarkers that may be useful in the diagnostic and prognostic classification of the studied sarcomas. The tumor-specific miRNA patterns we observed reflect underlying differences in cell lineage, differentiation state, and oncogenic pathways. While miR-9-5p is mainly oncogenic, miR-206 and miR-140-5p play versatile roles in non-neoplastic and neoplastic conditions, with their downregulation identified in several cancers.

The RMS miRNA profile included miR-206, miR-495-3p, miR-376-3c, miR-483, miR-450, and others that displayed significant differential expression in RMS compared with OS and EWS. miR-206 exhibited the best discriminatory power in differentiating RMS from the other tumors as confirmed by miRNA-ISH. This finding is supportive of another study which revealed higher serum levels of miR-206 in RMS [21]. Another study has suggested that miR-206 expression is muscle-specific and may vary with the extent of myogenic or rhabdomyoblastic differentiation of the tumor cells [22]. The expression of miR-206 in this study was seen across different RMS types, providing a useful marker of RMS regardless of the histologic type. miR-206 expression did not correlate with patient outcome (Figure 6). The lack of miR-140-5p expression in RMS further enhances the discriminatory value of miR-206, particularly by miRNA-ISH. Similarly, a combined miRNA signature (e.g., miR-135A-5p and miR-196A-5p expression with a lack of miR-let-7e expression by nCounter) could provide the strongest statistical power in discriminating ARMS from ERMS. Accurate RMS subclassification is important for guiding the appropriate treatment options and predicting the patient’s outcome. These findings underscore the importance of integrating miRNA biomarkers into a composite diagnostic metric to improve classification accuracy.

This study also highlighted distinct miRNA expression patterns in OS and EWS. Both tumors commonly arise in bone and differ in their biology and behavior. EWS presents as monomorphic small round cells while OS exhibits more pleomorphic cells with osteogenic differentiation. Thus, it is understandable that they share some overlapping but also distinct miRNA expression profiles. EWS was characterized by the significant expression of miR-9-5p, miR-29A-3p, miR-221-3p, miR-497-5p, miR-181a, miR-195-5p, and others. miR-9-5p was previously found to be overexpressed in EWS in comparison to normal cells and may regulate mesenchymal stem cell differentiation by suppressing osteogenic differentiation and promoting EWS tumorigenesis and phenotype [23,24]. The differential expression of miR-9-5p in EWS by nCounter and its decreased expression in OS and RMS was confirmed by miRNA-ISH experiments, which revealed a better predictive accuracy with an AUC value of 0.86 (Figure 4). This microRNA was also identified in metastatic EWS tumors and in OS patients with adverse outcomes, suggesting a role in tumor progression and aligning with previous studies that revealed that miR-9-5p is oncogenic and promotes cancer cell proliferation, invasion, and migration [24].

The low percentage of the miRNA-ISH hybridization signal with the miR-206 probe (Supplementary Table S1) reflects poor sensitivity and an inferior performance compared with the nCounter platform, which has clearly delineated its differential expression with better sensitivity. However, both methods have highlighted the specificity of this miRNA to RMS. Similarly, the low hybridization of miR-140-5p in OS reflects its lower discriminatory power with miRNA-ISH. It is worth mentioning that several EWS cases had high nCounter reading counts with miR-140-5p and positive miRNA-ISH signals, possibly due to the fact that this microRNA may be expressed by a subset of EWS with an aggressive course. According to nCounter, the expression of miR-140 was found to be associated with mortality in EWS, highlighting its prognostic significance and supporting a prior study linking its expression with lower overall survival in EWS and OS patients [25]. Previous studies have highlighted the role of miR-140-5p as a tumor suppressant, where it inhibits tumor growth and signaling related to cancer proliferation. Its expression in high-grade EWS and OS may be related to the upregulation of RUNX2 which induces cancer cell proliferation [25]. This miRNA is activated by OS tumor cells during treatment and may attenuate the efficacy of chemotherapy [26]. As stated earlier, miR-206 is not expressed in OS or EWS. This miRNA plays an important physiological role in bone development and the regulation of osteogenesis [27]. Thus, its lack of expression in OS is consistent with a tumor suppressive function, reinforcing previous studies associating decreased miR-206 expression with a high tumor grade, metastasis, and recurrence [28]. Other differentially expressed miRNAs include miR-199B and miR-214-3p that were implicated in OS tumorigenesis [29,30].

This study highlights the importance of miRNAs in understanding tumor biology and demonstrates the complementary utility of NanoString miRNA quantification methods and chromogenic miRNA-ISH in miRNA profiling and the analysis of FFPE sarcoma tissue samples. The NanoString nCounter platform has revealed itself as a robust sensitive screening tool for miRNA expression that is minimally impacted by formalin fixation and paraffin embedding. RNA extraction from FFPE tissue can be challenging due to crosslinking, fragmentation, and RNA degradation thus creating false negative/positive results in sequencing or amplification-based methods. However, NanoString nCounter does not require high-quality RNA input and can read from limited quantities and low RIN values without the need for amplification [31]. In a comparative study, nCounter outperformed RNA sequencing and microarray in detecting miRNA targets from FFPE tissue [32]. On the other hand, miRNA-ISH can provide specific probe results that are directly visualized by routine bright-field microscopy on slides that can be permanently archived. This enables correlation with tissue morphology and facilitates its integration into clinical practice and histology workflows. However, probe performance may be variable as miR-206 and miR-140-5p probes yielded fewer hybridization signals in RMS and OS (Supplementary Table S1) compared with NanoString nCounter, suggesting that miRNA-ISH sensitivity may be influenced by probe design, tissue quality, and fixation time. The opposite can be said about miR-9-5p, where it seems to perform with better sensitivity and specificity in miRNA-ISH than in nCounter. However, this statement cannot be substantiated as our TMA miRNA-ISH validation set included mixed adult and pediatric cases and a high number of EWS cases were excluded due to artifacts. This could have introduced heterogeneity and variability in miR-9-5p probe performance.

## 5. Conclusions

In conclusion, this study identified miR-206, miR-9-5p, and miR-140-5p as clinically relevant biomarkers for distinguishing pediatric sarcoma types and highlighted their potential prognostic value in identifying tumors with adverse outcomes. NanoString offers a sensitive screening method while targeted miRNA-ISH can easily be introduced in clinical labs. Combining miRNA profiles may yield better discriminatory and diagnostic utility. These distinct miRNA profiles may also provide insights into the oncogenesis of these rare tumors and could point to novel therapeutic strategies.

## Figures and Tables

**Table 1:** Number and distribution of tumors analyzed by NanoString nCounter in each demographic group.

Tumor Type	Osteosarcoma	Ewing’s Sarcoma	Rhabdomyosarcoma
**Age**			
<10	5	8	37
10–18	27	18	13
**Gender**			
Male	17	15	22
Female	15	11	28
**Ethnicity**			
Hispanic	22	15	43
African American	2	0	1
Asian	0	1	0
Caucasian	8	10	6
**Metastasis**			
Primary	15	14	24
Metastasis	17	12	26
**Outcome Status**			
Alive	13	9	32
Dead	19	13	18

**Table 2:** Differentially expressed miRNAs in each tumor using nCounter platform, when compared against the others, arranged according to level of statistical significance.

RMS Versus EWS (≥3-Fold Change, Adjusted *p* < 0.01)		RMS Versus OS (≥5-Fold Change, Adjusted *p* < 0.01)		EWS Versus OS (≥1.5-Fold Change, Adjusted *p* < 0.01)	
RMS	EWS	RMS	OS	EWS	OS
miR-206 *	miR-29A-3P	miR-206 *	miR-140-5P *	miR-9-5P *†	miR-214-3P
miR-1-3P	miR-9-5P *†	miR-495-3P	miR-218-5P	miR-29B-3P	miR-193A-5P+miR-193B-5P
miR-483-3P	miR-221-3P	miR-376C-3P	miR-181A-3P	miR-29A-3P	miR-199B-5P
miR-133A-3P	miR-29B-3P	miR-382-5P	miR-1469	miR-221-3P	miR-140-5P *
miR-433-3P	miR-196A-5P	miR-376A-3P	miR-140-3P	miR-376C-3P	miR-152-3P
miR-133B	miR-497-5P	miR-381-3P	miR-603	miR-195-5P	miR-199A-5P
miR-381-3P	miR-181A-3P †	miR-136-5P	miR-522-3P	miR-376A-3P	miR-450B-5P
miR-483-5P	miR-195-5P	miR-133A-3P	miR-3190-3P	miR-20A-5P+miR-20B-5P	miR-199A-3P+miR-199B-3P
miR-503-5P	miR-592	miR-1-3P	miR-551B-3P	miR-130A-3P	miR-548AR-5P
miR-495-3P	miR-222-3P	miR-127-3P	miR-1285-5P	miR-19B-3P	miR-579-3P
miR-323A-3P	miR-10B-5P	miR-660-5P	miR-147A	miR-106A-5P+miR-17-5p	miR-105-5P
miR-450A-5P	miR-221-5P		miR-1264		miR-574-5P
miR-135A-5P	miR-1972		miR-10A-5P		miR-31-5P
miR-432-5P	miR-490-3P		miR-548AR-5P		miR-218-5P
miR-299-5P	miR-491-5P		miR-193A-3P		miR-193A-3P
miR-431-5P	miR-873-5P		miR-3144-3P		miR-140-3P
miR-500A-5P+miR-501-5P	miR-1290		miR-3192-5P		miR-1233-3P
miR-660-5P	miR-150-5P		miR-571		miR-551B-3P
miR-376C-3P	miR-4284		miR-604		miR-146A-5P
miR-382-5P	miR-1285-5P		miR-515-3P		miR-574-3P
miR-409-3P	miR-181A-5P		miR-520A-5P		miR-708-5P
miR-335-5P	miR-630		miR-152-3P		miR-519D-3P
miR-136-5P	miR-494-3P		miR-574-5P		miR-155-5P
	miR-874-3P		miR-765		miR-365A-3P+miR-365B-3P
	miR-10A-5P		miR-1908-5P		miR-424-5P
	miR-1200		miR-4755-5P		miR-196B-5P
	miR-1246		miR-642A-5P		miR-450A-5P
	miR-593-3P		miR-3615		miR-28-5P
	miR-651-5P		miR-219A-2-3P		miR-503-5P
	miR-181C-5P		miR-1910-5P		miR-148A-3P
	miR-127-3P		miR-890		miR-28-3P
	miR-345-3P		miR-516B-5P		miR-23B-3P
	miR-505-3P		miR-589-5P		miR-455-5P
			miR-573		miR-24-3P
			miR-31-5P		miR-27B-3P
			miR-3928-3P		miR-21-5P
			miR-519B-5P+miR-519C-5P+miR-523-5P+miR-518E-5P+miR-522-5P+miR-519A-5P		
			miR-556-3P		
			miR-200C-3P		
			miR-891B		
			miR-579-3P		
			miR-504-3P		
			miR-566		
			miR-2053		
			miR-548J-3P		

* Confirmed by miRNA in situ hybridization. † Associated with metastasis in EWS.

**Table 3:** List of miRNAs differentially overexpressed in alveolar and embryonal rhabdomyosarcoma (RMS) (≥1.5-fold change, adjusted *p* < 0.05).

Alveolar RMS	Embryonal RMS
miR-362-5P	LET-7E-5P
miR-532-5P	miR-455-3P
miR-135-5P	miR-214-3P
miR-660-5P	miR-10B-5P
miR-500A-5P+miR-501-5P	miR-455-5P
miR-323A-3P	miR-199A-5P
	miR-99B-5P
	miR-196A-5P
	miR-218-5P
	miR-199A-3P+miR-199B-3P
	miR-130A-3P
	LET-7C-5P

## Data Availability

The raw data presented in this study are available upon request from the corresponding author. Raw data have been deposited in the NCBI Gene Expression Omnibus (GEO) public database under the file name: GEO_Nanostring_nCounter_Pediatric_Sarcomas.zip, available 11/24/2025 under accession number GSE310120.

## Supplementary Materials

The following supporting information can be downloaded at: https://www.mdpi.com/article/10.3390/cancers17233791/s1, Supplementary Table S1: miRNA probes used in miRNA in situ hybridization experiments and their staining results. Supplementary Table S2: nCounter reading counts of the three miRNAs analyzed in the tumors.

## References

[1] Bushati N., Cohen S.M. (2007). microRNA functions. Annu. Rev. Cell Dev. Biol..

[2] Lassandro G., Ciaccia L., Amoruso A., Palladino V., Palmieri V.V., Giordano P. (2021). Focus on MicroRNAs as Biomarker in Pediatric Diseases. Curr. Pharm. Des..

[3] Hata A., Lieberman J. (2015). Dysregulation of microRNA biogenesis and gene silencing in cancer. Sci. Signal..

[4] Lorio M.V., Croce C.M. (2012). MicroRNA dysregulation in cancer: Diagnostics, monitoring and therapeutics. A comprehensive review. EMBO Mol. Med..

[5] Lu J., Getz G., Miska E.A., Alvarez-Saavedra E., Lamb J., Peck D., Sweet-Cordero A., Ebert B.L., Mak R.H., Ferrando A.A. (2005). MicroRNA expression profiles classify human cancers. Nature.

[6] Saliminejad K., Khorram Khorshid H.R., Soleymani Fard S., Ghaffari S.H. (2019). An overview of microRNAs: Biology, functions, therapeutics, and analysis methods. J. Cell Physiol..

[7] Wei J.S., Johansson P., Chen Q.R., Song Y.K., Durinck S., Wen X., Cheuk A.T., Smith M.A., Houghton P., Morton C. (2009). microRNA profiling identifies cancer-specific and prognostic signatures in pediatric malignancies. Clin. Cancer Res..

[8] Crossland R.E., Macdonald J., Wang X.N. (2024). Assessing MicroRNA Profiles from Low Concentration Extracellular Vesicle RNA Utilizing NanoString nCounter Technology. Methods Mol. Biol..

[9] Wang F., Flanagan J., Su N., Wang L.C., Bui S., Nielson A., Wu X., Vo H.T., Ma X.J., Luo Y. (2012). RNAscope: A novel in situ RNA analysis platform for formalin-fixed, paraffin-embedded tissues. J. Mol. Diagn..

[10] Lin Y., Golovnina K., Chen Z.X., Lee H.N., Negron Y.L., Sultana H., Oliver B., Harbison S.T. (2016). Comparison of normalization and differential expression analyses using RNA-Seq data from 726 individual Drosophila melanogaster. BMC Genom..

[11] Ritchie M.E., Phipson B., Wu D., Hu Y., Law C.W., Shi W., Smyth G.K. (2015). limma powers differential expression analyses for RNA-sequencing and microarray studies. Nucleic Acids Res..

[12] Hennig C. Cran-Package Fpc. https://cran.r-project.org/web/packages/fpc/index.html.

[13] Ru Y., Kechris K.J., Tabakoff B., Hoffman P., Radcliffe R.A., Bowler R., Mahaffey S., Rossi S., Calin G.A., Bemis L. (2014). The multiMiR R package and database: Integration of microRNA-target interactions along with their disease and drug associations. Nucleic Acids Res..

[14] Lorenz R., Bernhart S.H., Zu Siederdissen C.H., Tafer H., Flamm C., Stadler P.F., Hofacker I.L. (2011). ViennaRNA Package 2.0. Algorithms Mol. Biol..

[15] Bankhead P., Loughrey M.B., Fernández J.A., Dombrowski Y., McArt D.G., Dunne P.D., McQuaid S., Gray R.T., Murray L.J., Coleman H.G. (2017). QuPath: Open source software for digital pathology image analysis. Sci. Rep..

[16] RStudio Team (2020). RStudio: Integrated Development for R. RStudio, PBC, Boston. http://www.rstudio.com.

[17] R Core Team (2021). R: A Language and Environment for Statistical Computing. https://www.R-project.org/.

[18] Wickham H., Averick M., Bryan J., Chang W., McGowan L.D., François R., Grolemund G., Hayes A., Henry L., Hester J. (2019). Welcome to the Tidyverse. J. Open Source Softw..

[19] Rota R., Ciarapica R., Giordano A., Miele L., Locatelli F. (2011). MicroRNAs in rhabdomyosarcoma: Pathogenetic implications and translational potentiality. Mol. Cancer.

[20] Veronez L.C., Fedatto P.F., Correa C.A.P., Lira R.C.P., Baroni M., da Silva K.R., Santos P., Antonio D.S.M., Queiroz R.P.S., Antonini S.R.R. (2022). MicroRNA expression profile predicts prognosis of pediatric adrenocortical tumors. Pediatr. Blood Cancer.

[21] Miyachi M., Tsuchiya K., Yoshida H., Yagyu S., Kikuchi K., Misawa A., Iehara T., Hosoi H. (2010). Circulating muscle-specific microRNA, miR-206, as a potential diagnostic marker for rhabdomyosarcoma. Biochem. Biophys. Res. Commun..

[22] Missiaglia E., Shepherd C.J., Patel S., Thway K., Pierron G., Pritchard-Jones K., Renard M., Sciot R., Rao P., Oberlin O. (2010). MicroRNA-206 expression levels correlate with clinical behaviour of rhabdomyosarcomas. Br. J. Cancer.

[23] Parafioriti A., Bason C., Armiraglio E., Calciano L., Daolio P.A., Berardocco M., Di Bernardo A., Colosimo A., Luksch R., Berardi A.C. (2016). Ewing’s Sarcoma: An Analysis of miRNA Expression Profiles and Target Genes in Paraffin-Embedded Primary Tumor Tissue. Int. J. Mol. Sci..

[24] He C., Liu M., Ding Q., Yang F., Xu T. (2022). Upregulated miR-9-5p inhibits osteogenic differentiation of bone marrow mesenchymal stem cells under high glucose treatment. J. Bone Miner. Metab..

[25] Green D., Singh A., Tippett V.L., Tattersall L., Shah K.M., Siachisumo C., Ward N.J., Thomas P., Carter S., Jeys L. (2023). YBX1-interacting small RNAs and *RUNX2* can be blocked in primary bone cancer using CADD522. J. Bone Oncol..

[26] Wei R., Cao G., Deng Z., Su J., Cai L. (2016). miR-140-5p attenuates chemotherapeutic drug-induced cell death by regulating autophagy through inositol 1,4,5-trisphosphate kinase 2 (IP3k2) in human osteosarcoma cells. Biosci. Rep..

[27] Guo S., Gu J., Ma J., Xu R., Wu Q., Meng L., Liu H., Li L., Xu Y. (2021). GATA4-driven miR-206-3p signatures control orofacial bone development by regulating osteogenic and osteoclastic activity. Theranostics.

[28] Zhang C., Yao C., Li H., Wang G., He X. (2014). Serum levels of microRNA-133b and microRNA-206 expression predict prognosis in patients with osteosarcoma. Int. J. Clin. Exp. Pathol..

[29] Chen Z., Zhao G., Zhang Y., Ma Y., Ding Y., Xu N. (2018). MiR-199b-5p promotes malignant progression of osteosarcoma by regulating HER2. J. Buon.

[30] Cai H., Miao M., Wang Z. (2018). miR-214-3p promotes the proliferation, migration and invasion of osteosarcoma cells by targeting CADM1. Oncol. Lett..

[31] Zheng C.M., Piao X.M., Byun Y.J., Song S.J., Kim S.K., Moon S.K., Choi Y.H., Kang H.W., Kim W.T., Kim Y.J. (2022). Study on the use of Nanostring nCounter to analyze RNA extracted from formalin-fixed-paraffin-embedded and fresh frozen bladder cancer tissues. Cancer Genet..

[32] Chilimoniuk J., Erol A., Rödiger S., Burdukiewicz M. (2024). Challenges and opportunities in processing NanoString nCounter data. Comput. Struct. Biotechnol. J..

