# Supplementary material for: MicroRNA Profiling Identifies Diagnostic and Prognostic Markers in Pediatric Sarcoma
Source: Cancers (Basel). 2025 Nov 27;17(23):3791. doi: 10.3390/cancers17233791 (PMC12691150; doi:10.3390/cancers17233791)
Supplement: Supplementary file 1 [file cancers-17-03791-s001.zip › cancers-3972834 Supplementary Table 1 edited.pdf]

Supplementary Table S1: miRNA probes used in miRNA in situ hybridization experiments and their staining results.

**Target**	**Condition**	**Vendor**	**Catalog No.**	**RMS**	**EWS**	**OS**
SR-RNU6-S1	Positive control	Advanced Cell Diagnostics	727871-S1	90/90 (100%)	14/14(100%)	42/42(100%)
SR-Scramble-S1	Negative control	Advanced Cell Diagnostics	727881-S1	0/90(0%)	0/14 (0%)	0/42 (0%)
SR-hsa-miR-206-S1	Experimental probe	Advanced Cell Diagnostics	1043221-S1	34/90 (38%)	0/14 (0%)	0/42 (0%)
SR-hsa-miR-9-5p	Experimental probe	Advanced Cell Diagnostics	1058691-S1	11/90 (12%)	12/14 (86%)	11/42 (26%)
SR-hsa-miR-140-5p	Experimental probe	Advanced Cell Diagnostics	728501-S1	2/90 (2%)	8/14 (57%)	20/42 (48%)
