# Supplementary material for: MicroRNA Profiling Identifies Diagnostic and Prognostic Markers in Pediatric Sarcoma
Source: Cancers (Basel). 2025 Nov 27;17(23):3791. doi: 10.3390/cancers17233791 (PMC12691150; doi:10.3390/cancers17233791)
Supplement: Supplementary file 1 [file cancers-17-03791-s001.zip › cancers-3972834 Supplementary Table 2 edited.pdf]

Supplementary Table S2: nCounter reading counts of the three miRNAs analyzed in the tumors

	miR-206	miR-9-5p	miR-140-5p
RMS	14.6224956100063	4.71067202339328	4.89411816448921
	12.9398390699378	2.73749259080308	5.95048631413728
	11.8953924011484	3.90103896428957	5.93078630768362
	10.155415800024	7.68077671420888	6.61745077884005
	17.4624871365279	4.07777082887851	6.53720244751581
	12.054245988285	9.87857370286532	6.7209729198036
	7.22234145408751	2.48087446768637	7.50324228071482
	11.8342556256242	7.07004749548429	6.42994344057067
	13.5969974117651	6.66439411786422	7.1713541065841
	12.8742126530426	9.55175812317734	6.09758222999153
	11.4586918714688	8.79572685874632	6.07674701005348
	13.4238523871118	8.95537171616513	6.19876159285266
	12.2651405940525	9.60623636802596	6.03469438306712
	14.2659252818987	8.58819453353874	6.0444803190851
	12.6039658382379	2.76476205014097	7.55048695622703
	14.2918819579785	5.91290854657173	5.8579420873538
	12.1606382141998	2.98936516950567	6.32234845290203
	14.2379737012162	9.23946446137843	6.12090785102653
	10.5924104790202	9.38656765173371	6.53337807595843
	12.7317499629992	5.2795879371211	6.2373597017318
	14.8717356699196	8.25127675670974	5.67113127228636
	14.0257014472793	8.98557187445166	5.8617432875988
	13.9488076251436	9.37019570472174	6.40672158074686
	12.252490943071	9.42533886589254	6.28134232944076
	15.5346036346929	7.05012328896363	5.82524987812342
	12.8846417405393	11.5209530760697	6.65973128203337
	10.7186702313088	7.36991834834573	6.06529126741557
	12.7669866549322	9.69703777736961	6.45602967786581
	13.8504823182107	9.80939414081344	6.2018356994944
	12.9667921463525	3.15140885053891	7.28069186748388
	14.3077665735647	1.61415516533931	5.68944329264355
	12.7298646897504	2.87260139385369	5.77949198946221
	18.6170960693041	2.95112178533042	5.95112178533042
	9.87039596669779	1.91184325126678	5.76982424639435
	12.5688984600425	9.02644938660403	5.56439706780759
	12.7224747950902	9.52896448551873	5.80951665261055
	12.3563627880058	6.79235990592029	6.05127820328186
	13.2144802696748	3.41196390455354	5.99692640527469
	13.4457589512663	9.33625445718416	5.96044612157084
	12.9994225236392	10.8306721831754	6.19187956703705

	15.8219798124224	7.81633005267308	6.05467757965541
	12.0074525002256	9.74360047179083	6.35128304901207
	15.3229535559675	7.9194194583932	5.72540883842658
	11.9066921063188	4.85140967081758	6.35836965953747
	13.3311961481266	8.77504403470489	5.87274070859202
	14.6266680124993	8.87767266949351	7.21835472444623
	14.2940013299328	3.84531141112436	5.99731450456941
	12.8364000545625	10.4339307242068	6.48858780029292
	14.2704691556533	3.44516960760147	5.92321690440612
	13.7269281252798	7.79988415856101	6.32938038242477
EWS	6.50517118014887	11.2732294448059	6.87838850373308
	6.42160285402988	7.78565642458992	7.0242673564845
	4.04496756907875	10.7518664379428	6.86599742803343
	9.39864179883904	11.2872776690181	6.00240400668894
	11.1485545927581	6.84112606756583	6.51919797267846
	3.4644213247497	9.1311779166345	6.69708208153997
	4.127112310151	11.207485726615	6.97510921670595
	7.37129861864512	9.39877935506722	6.25582140122518
	4.49430849109352	11.9872066400157	6.95943122180867
	5.2064808368327	12.4173260935883	4.98408841549626
	9.69512336416643	8.15080284794262	7.88776844210883
	14.0823998091452	10.1589304720464	6.54022775949998
	7.80072701742876	12.3440742044458	6.99182330827567
	3.42969252172336	12.5250895445159	7.2684116146903
	9.19412588686655	12.2911960557909	6.40592206515386
	6.21651123119159	14.0905248810563	7.34426677838997
	3.56925536315383	6.75907992203385	7.19861198323344
	3.39589850155681	11.4810863961478	6.2889832976403
	5.93668631766773	11.0183752216314	6.57411623828303
	4.45100063167771	8.15795365677747	7.85539088675704
	5.11602503482434	10.6032908616584	6.7009875355455
	13.8074239824993	8.32044063640414	6.81596805354335
	6.31564379403329	9.81061439387441	7.67039051411878
	3.83530165560381	10.6602603961323	7.37446046671184
	3.95494071976847	11.5299822967782	7.3143435200745
	5.69916290399258	5.79227230838406	7.92155532532903
OS	9.04891116055863	7.46394865983748	7.46394865983748
	4.29464475333077	4.51703717466722	10.7375882491795
	13.5616629285977	4.69129820901425	8.15072982765155
	6.49365415329376	4.1717260584064	7.75668855912755
	5.12328358180718	2.53832108108602	7.58271520044448
	11.125375928794	1.69701575608968	10.4914316224398
	12.2497216853512	4.94594093717412	8.94594093717412
	12.2867620606766	4.83280557210542	11.5309314244083

	5.12330115723783	5.4452292521252	9.80980168442105
	9.37166710321383	7.28420426196349	7.98464398010459
	9.70258795114132	5.56836701138069	7.61101134878918
	8.20314926331487	4.67958730725786	7.38002702539895
	8.59637677281308	4.84148927064961	13.3009208892869
	10.3002765661037	7.13035156466141	7.13035156466141
	5.25613026363092	4.38166114571478	7.22296339969572
	8.16139613832638	4.35404121626877	10.7634321524065
	3.9440879174276	5.97971182715832	8.09789325350664
	4.63547228564004	9.54876065244671	10.6548776848126
	4.47274966702834	9.941984460696	11.5061726685658
	11.587064135716	7.07926949551728	8.77970921365838
	7.02345698601235	5.35360558770468	9.35360558770468
	3.20546059932961	9.70264713996233	8.62331311421551
	2.78978999921999	10.2132561205694	5.92364574595478
	10.0996251312011	5.55530461497728	6.55530461497728
	7.02800275287814	7.73427154982143	11.6097979745206
	6.414806134802	9.64131466461068	9.7482298685272
	7.04836129063898	9.30774791945763	11.3702893855263
	5.04611521741413	8.15045187722887	11.6240475160678
	4.34901327931159	4.17908827786928	7.40790696836516
	9.55575387406098	6.19820186944289	8.72176382549991
	5.06344142734566	7.64840392806682	9.150904268596
	4.29361509846604	5.76754628679845	8.15711309856117
